# Syngeneically transplanted insulin producing cells differentiated from adipose derived stem cells undergo delayed damage by autoimmune responses in NOD mice

**DOI:** 10.1038/s41598-022-09838-x

**Published:** 2022-04-07

**Authors:** Kazunori Tokuda, Tetsuya Ikemoto, Shoko Yamashita, Katsuki Miyazaki, Shohei Okikawa, Shinichiro Yamada, Yu Saito, Yuji Morine, Mitsuo Shimada

**Affiliations:** Department of Digestive and Transplant Surgery, Tokushima University, Tokushima, 770-8503 Japan

**Keywords:** Mesenchymal stem cells, Type 1 diabetes, Autoimmunity

## Abstract

Insulin-producing cells (IPCs) generated by our established protocol have reached the non-clinical ‘proof of concept’ stage. Our strategy for their clinical application is the autotransplantation of IPCs into patients with type 1 diabetes mellitus (T1DM). In this context, the autoimmunity that characterized T1DM is important, rather than allorejection. We aimed to determine how these IPCs respond to T1DM autoimmunity. IPCs were generated from the subcutaneous fat tissue of non-obese diabetic (NOD) mice using our protocol. IPCs derived from NOD mice were transplanted under the kidney capsules of NOD mice at the onset of diabetes and the subsequent changes in blood glucose concentration were characterized. Blood glucose decreased within 30 days of transplantation, but increased again after 40–60 days in three of four recipient NOD mice. In tissue samples, the numbers of CD4^+^ and CD8^+^ T cells were significantly higher 60 days after transplantation than 30 days after transplantation. In conclusion, IPCs significantly ameliorate the diabetes of mice in the short term, but are damaged by autoimmunity in the longer term, as evidenced by local T cells accumulation. This study provides new insights into potential stem cell therapies for T1DM.

## Introduction

Type 1 diabetes mellitus (T1DM) is a chronic autoimmune disease characterized by insulitis and complete insulin deficiency owing to the destruction of pancreatic β-cells. The incidence of T1DM has been increasing worldwide for several decades^1^. Islet transplantation is one of the major therapies for T1DM; however, there is an urgent need for the development of new cell sources in countries such as Japan, which suffers from a severe shortage of donors. One of the solutions is regenerative medicine, such as the generation of insulin-producing cells (IPCs).

Adipose-derived stem cells (ADSCs) have been used in several studies. They have been studied as a potential new source of cells, not only because of their trophic effects, but also because of their multipotency^2,3^. Moreover, they can be collected from a donor’s fat tissue using a technique that is less invasive than the methods required to harvest other mesenchymal stem cells (MSCs), such as bone marrow-derived stem cells, and which is therefore associated with fewer ethical issues. In addition, it is easier to obtain a larger yield of ADSCs than other types of MSC^4^. Therefore, the production of IPCs by differentiating ADSCs has potential in the establishment of a new treatment for T1DM.

We have previously reported the generation of functional IPCs from ADSCs^5^ with three-dimensional (3D) culture and a xenoantigen-free protocol^6–10^. The quality and functionality of IPCs generated in this way have been assessed using techniques such as the stimulation index (SI), which can be used to evaluate autonomous glucose response ability, and showed that the cells have satisfactory functionality for clinical use^5–10^. Moreover, we reported that 120 days after transplantation, IPCs showed almost no central necrosis and high expression of vascular endothelial factor (VEGF), and demonstrated that blood vessels penetrated the IPC clusters. Our protocol has reached the non-clinical ‘proof of concept’ stage under the regulation of the Japanese Pharmaceuticals and Medical Devices Agency (AMED). This study provided new insights into stem cell therapy for T1DM and may lead to a first-in-human clinical trial to solve the severe donor shortage. However, there are still several scientific questions that need to be elucidated before considering our protocol for a first-in-human clinical trial.

We have succeeded in generating IPCs from ADSCs isolated from the adipose tissue of a patient with T1DM^9^. However, we need to determine whether these IPCs are susceptible to autoimmune attack after transplantation into patients with T1DM. We previously reported that single transplantation of 2.0 × 10^6^ IPCs generated from human subcutaneous fat tissue under the kidney capsule of a nude mouse normalizes its blood glucose (BG) concentration for up to 120 days^6^. However, it is unknown whether newly generated IPCs would be subsequently damaged by the autoimmunity that characterizes T1DM. Furthermore, if these IPCs are not damaged in this way, it is unknown what their fate would be.

The mechanism of islet destruction in T1DM is still under investigation. β-cell destruction causes a reduction in insulin secretion, the development of hyperglycemia, and ultimately clinical symptoms in patients^11,12^. It is widely accepted that T1DM is associated with the activation of CD4^+^ T cells that recognize islet autoantigens and cytotoxic CD8^+^ T cells that attack β-cells. It has been reported that various autoantigens are associated with T1DM, including insulin, islet-cell autoantigen (ICA), the 65-kDa isoform of glutamic acid decarboxylase (GAD65), and zinc transporter 8 (ZnT8)^13–15^. These autoantigens are recognized by antigen-presenting cells and CD4^+^ T cells in the peripheral circulation, and then CD8^+^ T cells infiltrate and destroy the islets^12^. Furthermore, the involvement in T1DM of programmed cell death ligand 1 (PD-L1), a well-known immune checkpoint, has been reported^16^. PD-L1 plays a major role in suppressing acquired immunity in autoimmune diseases, allogeneic transplantation and a stem cell experiment^17^. We have focused on the expression of PD-L1 in our IPCs, and found that PD-L1 expression may limit the effect of autoimmunity in IPCs^9^.

The non-obese diabetic (NOD) mouse, a model of T1DM that was established in the 1970s, is an important tool for investigations of the mechanisms of T1DM^18–20^. In NOD mice, autoreactive T cells recognize specific islet-related autoantigens such as insulin, GAD65, and ZnT8, leading to the destruction of β-cells. NOD mice, unlike other animal models that are studied as part of autoimmunity research, develop a spontaneous disease similar to human T1DM^19^. Our strategy for the clinical application of IPCs is autotransplantation into patients with T1DM. Therefore, the autoimmunity that characterizes T1DM is the primary concern, rather than allorejection. In the present study, we isolated ADSCs from the subcutaneous fat tissue of NOD mice and differentiated them into IPCs using our established protocol. To elucidate whether the generated auto-IPCs are damaged by autoimmunity, we measured the expression of major islet-specific autoantigens and PD-L1 in IPCs derived from NOD mice (NOD-IPCs) and characterized the function and fate of NOD-IPCs when transplanted into NOD mice as a syngeneic model of our strategy.

## Results

### Characteristics of ADSCs isolated from the subcutaneous fat tissue of NOD mice

**Figure 1:**
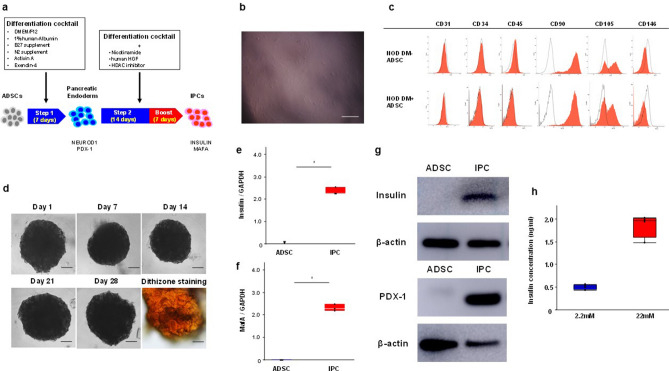
Quality of the generated NOD-IPCs. (**a**) Schema of our differentiation protocol for the generation of NOD-IPCs from ADSCs. (**b**) Morphology of ADSCs isolated from the fat tissue of NOD mice. Scale bar, 100 μm. (**c**) FACS analysis of ADSCs isolated from the fat tissue of NOD mice. NOD DM- ADSCs: ADSCs isolated from normoglycemic NOD mice. NOD DM + ADSCs: ADSCs isolated from diabetic NOD mice. ADSCs were positive for CD90 and CD105, and negative for CD31, CD34, CD45 and CD146. (**d**) Morphology of a NOD-IPC cluster on days 1, 7, 14, 21, and 28. NOD-IPC clusters formed sphere-like structures within 24 h of the start of the 3D culture, and their morphology was maintained for at least 28 days. NOD-IPCs showed strong staining with dithizone on day 28. Scale bar, 200 μm. (**e, f**) mRNA expression of *Insulin* and *MafA* in the generated NOD-IPCs on day 28. The lines in the boxes show the median values (*P < 0.05, Mann–Whitney *U* test). (**g**) Western blots for insulin and PDX-1 in ADSCs and NOD-IPCs. The gels/blots were cropped from different parts of the different gels and grouped. The original blots/gels are presented in Supplementary Fig. 1. (**h**) Glucose-stimulated insulin secretion by NOD-IPCs. The mean insulin secretion by cells cultured in basal 2.2 mM glucose-containing medium was 0.50 ng/mL and that by cells cultured in 22 mM glucose-containing medium was 1.86 ng/mL. The SI for the NOD-IPCs was 3.7.

ADSCs isolated from the subcutaneous fat tissue of NOD mice were passaged two-to-three times prior to use. They were spindle-shaped (Fig. 1b) and fluorescent-activated cell sorting (FACS) analysis demonstrated that the isolated and passaged ADSCs were positive for the mesenchymal markers CD90 and CD105 and negative with respect to CD31, CD34, CD45 and CD146 expression (Fig. 1c).

### The generated NOD-IPCs show appropriate characteristics of β-cells

NOD-IPCs developed a sphere-like cell cluster formation within 24 h of the start of 3D culture, and this morphology was maintained until day 28. The NOD-IPCs demonstrated strong dithizone staining at day 28 (Fig. 1d). In the Image J analysis, the dithizone staining intensity of NOD-IPCs reached 241 (Fig. 1d). The staining intensity of these NOD-IPCs was very similar to the reported intensity of isolated fresh human islets (the intensity; 244)^8^. The mRNA expression of *Insulin* and *MafA* in the generated NOD-IPCs on day 28 was significantly higher than that in ADSCs (both *P* < 0.05, Mann–Whitney *U* test, Fig. 1e, f). In addition, the expression of insulin and pancreatic and duodenal homeobox 1 (insulin promotor factor 1; PDX-1) proteins in ADSCs and NOD-IPCs was evaluated by western blotting. Although neither were expressed in ADSCs, both were strongly expressed in NOD-IPCs (Fig. 1g). Furthermore, we quantified glucose-stimulated insulin secretion by NOD-IPCs, and found that the mean basal (2.2 mM) and glucose-stimulated (22 mM) insulin secretion by the cells was 0.50 ng/mL and 1.86 ng/mL, respectively, corresponding to a SI of 3.7 (Fig. 1h).

### Immunofluorescence staining of the generated NOD-IPCs

**Figure 2:**
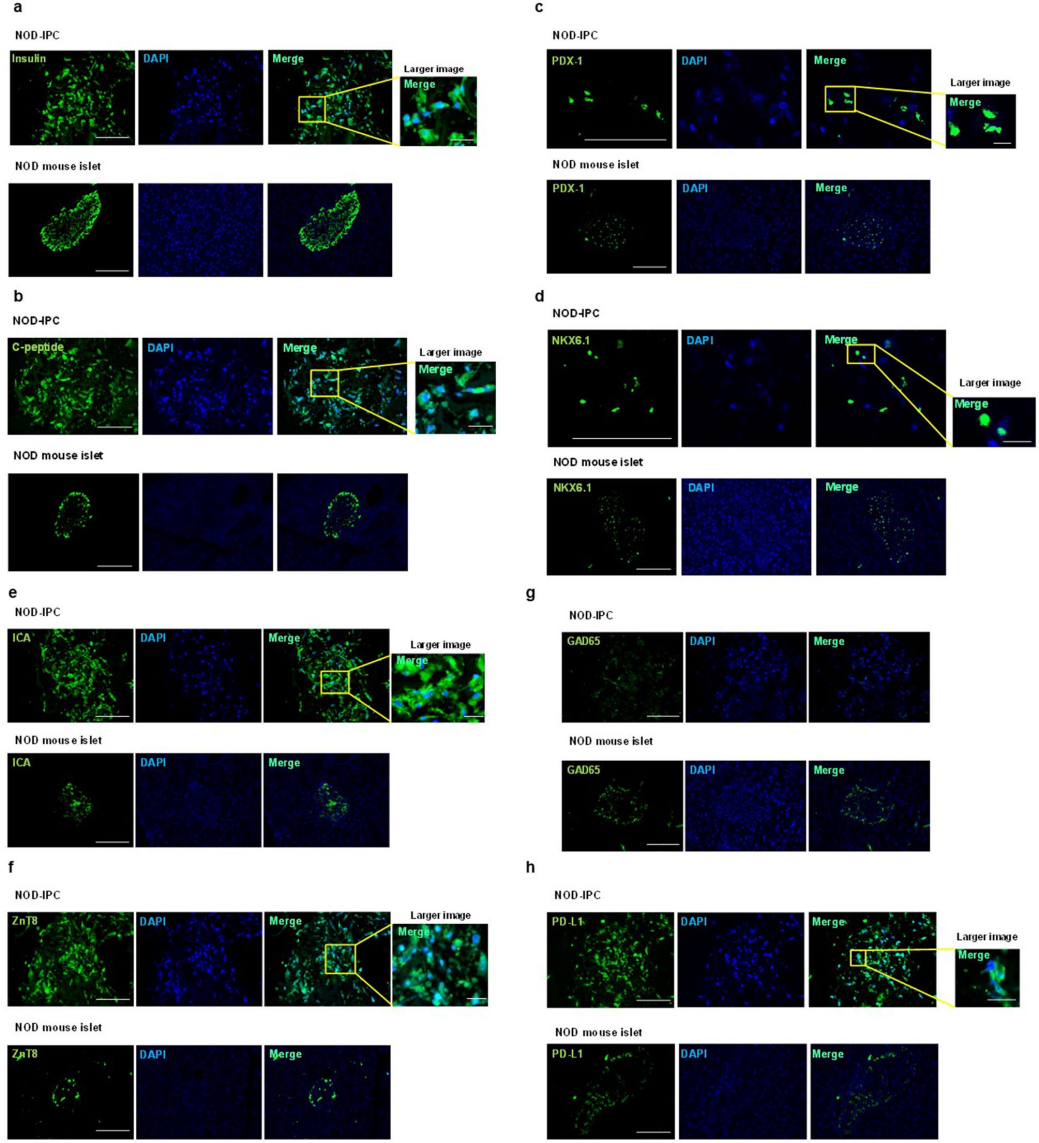
Morphology of the generated NOD-IPCs. (**a–d**) Immunofluorescence staining of insulin (**a**), C-peptide (**b**), PDX-1 (**c**), and NKX6.1 (**d**) in NOD-IPCs on day 28. Green: insulin, C-peptide, PDX-1, or NKX6.1; blue: DAPI. Scale bar, 200 μm; scale bar of the larger image, 25 μm. (**e–g**) Immunofluorescence staining of autoantigens (ICA, GAD65, and ZnT8) in NOD-IPCs on day 28. ICA and ZnT8 were highly expressed, whereas GAD65 was not expressed. Green: ICA, ZnT8, or GAD65; blue: DAPI. Scale bar, 200 μm; scale bar of the larger image, 25 μm. (**h**) Immunofluorescence staining of PD-L1, which was highly expressed in NOD-IPCs. Green: PD-L1; blue: DAPI. Scale bar, 200 μm; scale bar of the larger image, 25 μm. Islets of NOD mice were used as a control.

Immunofluorescence staining of NOD-IPCs for insulin, C-peptide, PDX-1 and NKX6.1 showed high expression of each on day 28 (Fig. 2a–d). To determine whether islet autoantigens were expressed in NOD-IPCs, immunofluorescence staining for ICA, GAD65, and ZnT8 was also performed. This showed strong expression of ICA and ZnT8, but extremely low expression of GAD65 (Fig. 2e–g). Next, we assessed the expression of PD-L1, which plays an important role in autoimmune diseases, and immunostaining also revealed high expression of PD-L1 (Fig. 2h). Islets of NOD mice were used as a control.

### NOD-IPC transplantation normalizes the glycemia of NOD mice

**Figure 3:**
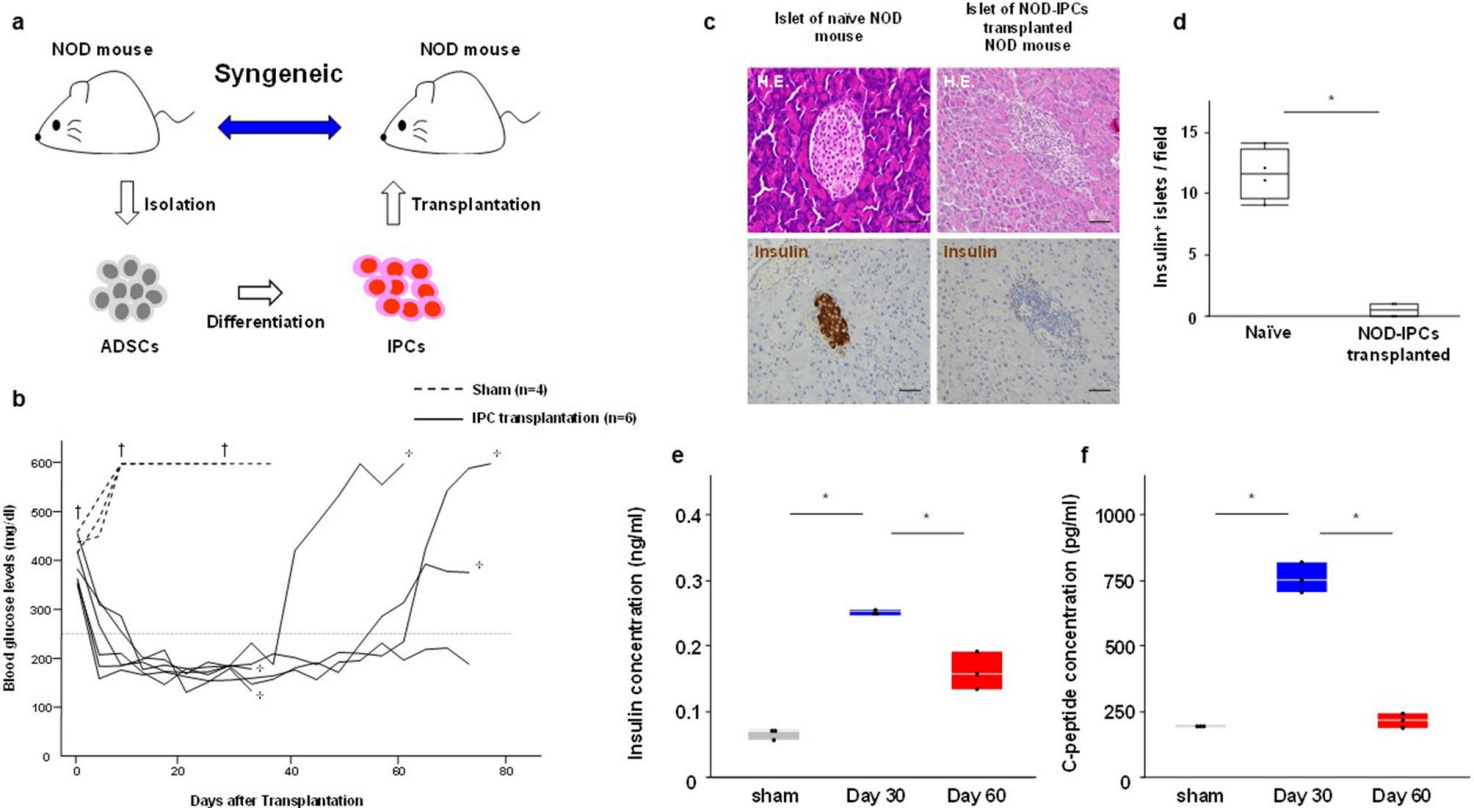
NOD-IPC transplantation. (**a**) Syngeneic model of NOD-IPCs transplantation. (**b**) Blood glucose (BG) concentration profile of NOD mice at the onset of diabetes after NOD-IPC transplantation. In the sham group (*n* = 4), the BG concentration remained high. However, the BG concentration gradually decreased after NOD-IPC transplantation to < 200 mg/dL at 10 days post-transplantation and stayed low until 30 days post-transplantation (*n* = 6). In three out of four recipient NOD mice, the BG concentration, which was improved by NOD-IPC transplantation, gradually increased to > 250 mg/dL 40–60 days after transplantation. †: death. ✢: killed. (**c**) The islets of diabetic NOD mice transplanted with NOD-IPCs were not stained by the anti-insulin antibody. NOD mice that did not develop diabetes were used as controls. H&E staining. Scale bar, 100 μm. (**d**) The number of insulin-positive islets was significantly lower in NOD-IPC-transplanted NOD mice than in naïve NOD mice. The lines in the boxes show the median values (*P < 0.05, Mann–Whitney *U* test). (**e**) Serum insulin concentration in NOD mice transplanted with NOD-IPCs. The lines in the boxes show the median values (*P < 0.05, one-way ANOVA followed by the Tukey–Kramer test). (**f**) Serum C-peptide concentration in NOD mice transplanted with NOD-IPCs. The lines in the boxes show the median values (*P < 0.05, one-way ANOVA followed by the Tukey–Kramer test).

To determine whether NOD-IPCs would likely be damaged by autoimmunity when transplanted into recipients with T1DM, we transplanted NOD-IPCs into NOD mice at the onset of diabetes (Fig. 3a). The BG profile of the NOD mice after NOD-IPC transplantation is shown in Fig. 3b. Their BG concentrations gradually decreased after transplantation to below 200 mg/dL at approximately 10 days post-transplantation and remained at that level until 30 days post-transplantation (*n* = 6). Two mice were killed for pathological investigations approximately 30 days after transplantation. The BG concentrations of three out of the remaining four recipient NOD mice (75%) gradually increased again to > 250 mg/dL 40–60 days after transplantation. According to previous reports of islet transplantation in NOD mice, recipients became normoglycemia about 3 days after transplantation; however, 1 to 2 weeks after transplantation, the islet grafts were infiltrated with lymphocytes and disrupted, and the recipients returned to a hyperglycemic state^21–23^. The islets of the diabetic NOD mice that had been transplanted with NOD-IPCs did not show staining after application of the anti-insulin antibody 30 days after transplantation (Fig. 3c). Furthermore, the number of insulin-positive islets in the pancreas was significantly lower in the NOD-IPC-transplanted NOD mice than in naïve NOD mice (*P* < 0.05, Mann–Whitney *U* test, Fig. 3d). The serum insulin and C-peptide concentrations of the NOD mice 30 days after transplantation were significantly higher than those of the sham group (*P* < 0.05, Mann–Whitney *U* test), but they were significantly lower after approximately 60 days (*P* < 0.05, Mann–Whitney *U* test, Fig. 3e, f).

### Pathology of transplanted NOD-IPCs

**Figure 4:**
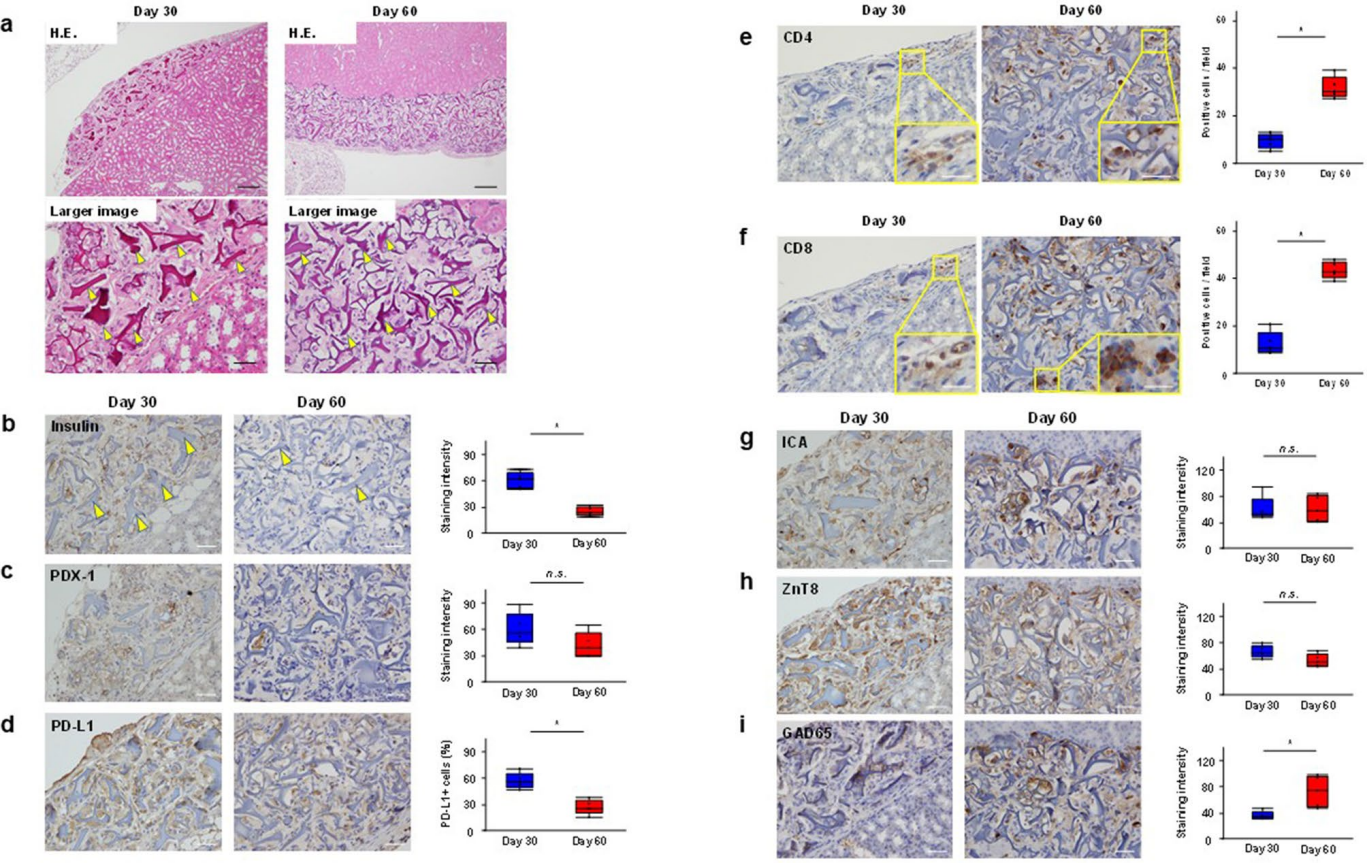
Pathological examination of the transplanted NOD-IPCs. (**a**) H&E-stained transplanted NOD-IPCs were examined under the kidney capsule. RCP μ-pieces were identified at the indicated time points (yellow arrowheads). Upper panels. Original magnification, × 100. Scale bar, 200 μm. Lower panels. Original magnification, × 400. Scale bar, 50 μm. (**b–d**) Immunohistochemical staining of the NOD-IPCs. These cells showed substantial staining by the anti-insulin and anti-PDX-1 antibodies on Day 30; however, they were barely stained on Day 60. The expression of PD-L1 was maintained in the transplanted NOD-IPCs on Day 30; however, the expression was significantly lower on Day 60. Original magnification, × 400. Scale bar, 50 μm. (**e, f**) On Day 30, small numbers of CD4^+^ and CD8^+^ T cells were observed around the transplanted NOD-IPCs. However, many CD4^+^ and CD8^+^ T cells had accumulated around the transplanted NOD-IPCs and infiltrated deeply into the NOD-IPCs clusters by Day 60. The numbers of CD4^+^ and CD8^+^ T cells were significantly higher on Day 60 than on Day 30. Original magnification, × 400. Scale bar, 25 μm. The lines in the boxes show the median values (*P < 0.05, Mann–Whitney *U* test). (**g–i**) The expression of ICA and ZnT8, which were expressed before transplantation, was maintained after transplantation (no difference in staining intensity between the time points). Conversely, GAD65, which was not expressed in vitro, was expressed in NOD-IPCs after transplantation. Moreover, the staining intensity was significantly higher on Day 60 than on Day 30. Original magnification, × 400. Scale bar, 50 μm. The lines in the boxes show the median values (*P < 0.05, Mann–Whitney *U* test).

At 30 days after transplantation, when BG levels were normalized (Day 30), or 60 days after transplantation, when BG levels were once again hyperglycemic (Day 60), the recipients were killed. Kidneys transplanted with NOD-IPCs were removed at each time point. Hematoxylin and eosin (H&E)-staining of the transplanted NOD-IPCs in their location beneath the kidney capsule revealed pathological micro-pieces of recombinant protein (RCP μ-pieces) at both time points (Fig. 4a). As we have previously reported^6,7^, a mixture of RCP μ-pieces and NOD-IPCs were present around the transplanted kidney. Continuous sections were used for immunohistochemical staining in Fig. 4. The NOD-IPCs appeared morphologically normal and immunohistochemical staining showed high levels of expression of both insulin and PDX-1 on day 30, but very little expression on Day 60 (significantly lower staining intensity: *P* < 0.05, Mann–Whitney *U* test, Fig. 4b, c). In addition, there was substantial expression of PD-L1 in the transplanted NOD-IPCs at Day 30, but the expression was lower on Day 60, with the percentage of PD-L1^+^ NOD-IPCs on Day 60 being significantly lower than on Day 30 (*P* < 0.05, Mann–Whitney *U* test, Fig. 4d).

To determine whether the transplanted NOD-IPCs had been damaged by autoimmune responses, we performed immunohistochemical staining with anti-CD4 and anti-CD8 antibodies. On Day 30, small numbers of CD4^+^ and CD8^+^ T cells were observed around the transplanted NOD-IPCs. However, many CD4^+^ and CD8^+^ T cells gathered around the transplanted NOD-IPCs and infiltrated deep into the clusters on Day 60. Namely, CD4^+^ and CD8^+^ T cells were significantly increased on Day 60 compared with on Day 30 (*P* < 0.05, Mann–Whitney *U* test, Fig. 4e, f). ICA and ZnT8 expression, which were expressed before transplantation, was maintained after transplantation and the immunohistochemical staining intensity of each was similar at the two time points (Fig. 4g, h). Conversely, GAD65, which was not expressed in vitro, was expressed in NOD-IPCs after transplantation, and the corresponding staining intensity was significantly higher on Day 60 than on Day 30 (Fig. 4i).

## Discussion

T1DM is an autoimmune disease in which the immune system attacks β-cells, and both adaptive and innate immunity are involved in the progression of T1DM^24^. The pathophysiology of T1DM involves the islet-specific activation and proliferation of autoreactive CD4^+^ and CD8^+^ T cells, which results in progressive β-cell destruction^19^. ADSCs have a trophic effect in addition to being multipotent; however, the administration of ADSCs did not ameliorate T1DM in clinical trials. Moreover, the use of ADSCs is associated with the risk of malignant neoplasm formation. We have previously reported that IPCs do not differentiate into other undesirable cell types and do not show increased DNA content. Furthermore, no macroscopic teratomas or carcinomas were observed at transplantation sites 120 days after the procedure in a murine model^6,7^. Therefore, from the viewpoint of efficacy and tumorigenicity, it would be more effective and safer to differentiate ADSCs into IPCs before transplantation than to directly transplant ADSCs. Our strategy for clinical application is to collect subcutaneous fat tissue from a patient with T1DM under local anesthesia, isolate ADSCs at a Cell Processing Center (CPC) according to the Good Gene, Cellular, and Tissue-based products Manufacturing Practice (GCTP) criteria, differentiate ADSCs into IPCs using the already established mass culture method (which cannot be disclosed because it is intellectual property), and finally autotransplant the generated IPCs. The quality of this method has already been approved by the Pharmaceuticals and Medical Devices Agency (PMDA), which is a respected Japanese authority; thus, we are currently preparing for an investigator initiated clinical trial. Assuming that this strategy would be used in a man in his twenties who is in Japan and has T1DM, and comparing the cost of this procedure with those of other treatments, such as lifelong insulin administration or islet transplantation with lifelong immunosuppressive therapy, although our strategy is expensive to implement, it would be more cost effective in the long term (insulin therapy: USD 619,199; islet transplantation: USD 958,134, and IPCs auto-transplantation: USD 396,353; costs calculated with a diabetologist; data submitted to a journal).

We aim to establish an investigator-initiated clinical trial of the autotransplantation strategy using the described IPC transplantation in the future. Because autotransplantation is not associated with allorejection, the patients should not require immunosuppression. However, it is unclear whether IPCs that are newly generated from ADSCs would be recognized as “β-cells” as part of the autoimmune responses in patients with T1DM. According to previous reports of islet transplantation in NOD mice, recipients became normoglycemia about 3 days after transplantation; however, 1 to 2 weeks after transplantation, the islet grafts were infiltrated with lymphocytes and disrupted, and the recipients returned to a hyperglycemic state^21–23^. It is well known that islet grafts are attacked by T1DM autoimmunity in NOD mice, but it is unknown as to what happens to syngeneic-transplanted insulin-producing cells. To our knowledge, there have been no studies of whether IPCs generated from ADSCs are affected by the autoimmunity that characterizes T1DM. It has been reported that MSCs have an immunoregulatory effect^25,26^; therefore, IPCs derived from MSCs may also demonstrate immunoregulatory effects. Furthermore, because our IPCs are newly generated cells, we hypothesized that these cells may escape the autoimmune damage caused to β-cells. We have previously reported that PD-L1, the expression of which is considered to represent immunological escape, is expressed on IPCs^9^, which suggests that IPCs may be able to temporarily escape autoimmunity. However, they may be subject to damage by autoimmune responses in the longer term via an unknown mechanism.

The purpose of this study was to investigate how the IPCs generated by our protocol respond to the autoimmune responses of patients with T1DM. First, we succeeded in generating IPCs with good functionality using NOD mice, which are a model of T1DM. The SIs of the generated NOD-IPCs were comparable to those of previously reported human IPCs^5–10^. We previously reported that electron microscopy revealed that the nuclei of the IPCs contained dense cystic microstructures and organelles such as rough endoplasmic reticulum and mitochondria, and these dense cystic microstructures in the generated IPCs morphologically resembled insulin secretion granules observed in normal islets from naïve rat pancreas as a characteristic analysis^6,8^. Moreover, we described various positive markers, such as dithizone, C-peptide, insulin and PDX-1 morphological markers, and *INS*, *MAFA*, *SLC39A4*, and *SLC30A8* mRNA expression. Thus, in this study, we performed immunofluorescence staining of NKX6.1 as an additional characterization of IPCs, using mouse islets as a positive control. Various islet-related autoantigens that are targeted by T cells have been reported^27–29^, and we found that islet-related autoantigens such as ICA and ZnT8, are expressed in these NOD-IPCs. Therefore, it was likely that the NOD-IPCs would be attacked and destroyed by T cells after transplantation, in addition to native β-cells. However, PD-L1 was also highly expressed in NOD-IPCs. The expression of this immune checkpoint leads to immunological evasion, and there have been some reports that PD-L1 is involved in the protection of β-cells and delays the onset of T1DM^30–34^. Wang et al.^34^ reported that transgenic overexpression of PD-L1 in the pancreatic β-cells of NOD mice protect β-cells by reducing the number of diabetogenic T cells. Moreover, it has been shown that immune checkpoint inhibitors induce T1DM^35–38^, all of which emphasizes that PD-L1 is important for the onset of T1DM.

It is very important to determine whether these IPCs express autoantigens at the same level as native β-cells in patients with T1DM. To address this question, we transplanted NOD-IPCs into NOD mice as a syngeneic model and found that these IPCs had resolved the hyperglycemic state 30 days after transplantation, but that over a longer period (60 days after transplantation) they were subject to damage by T cells. These results imply that newly generated IPCs would alleviate the symptoms of T1DM, but that they would eventually be recognized and destroyed by T1DM autoimmune responses. Thus, a further therapeutic intervention is necessary to improve the long-term survival of such transplanted IPCs.

One possibility is that PD-L1 expression in IPCs might protect transplanted IPCs as it does β-cells. Colli et al. reported that PD-L1 expressed in β-cells is upregulated by interferon (IFN)-α and γ via interferon regulatory factor 1 (IRF1)^39^. Consistent with this, Osum et al. reported that β-cells suppress the effects of autoreactive T cells by expressing PD-L1 in response to IFN-γ. However, PD-L1 alone was not sufficient to prevent autoimmune-induced damage; therefore, combination approaches should be considered^40^. Moreover, many researchers have reported alternative methods of protecting β cells. Weng et al. showed that the signal transducer and activator of transcription 3 (STAT3)-phosphatase and tensin homolog (PTEN)-AKT pathway is an important regulator of β-cell function and apoptosis^41^. They also suggested that interventions targeting this signaling pathway, such as inhibition of PTEN activity, may represent a new therapeutic strategy^41^.

Palmitic acid esters of hydroxystearic acid (PAHSAs) are endogenous lipids and the most studied fatty acid esters of hydroxyl-fatty acid (FAHFAs), and have been reported to improve insulin sensitivity and reduce insulin resistance^42^. Syed et al.^43,44^ demonstrated that PAHSAs weaken autoimmune responses by suppressing the activation and infiltration of T cells, and increase the viability of β cells by reducing the endoplasmic reticulum stress response through suppression of the c-Jun N-terminal kinase (JNK)/mitogen-activated protein kinase (MAPK) pathways. It has also been reported that γ-aminobutyric acid (GABA) is involved in the inhibition of inflammatory T cells and the replication and survival of β cells^45,46^. We plan to further investigate the protective effects of these β-cell protective agents in the IPCs described herein.

It has also been reported that the outcomes of transplantation are improved by transplanting MSCs at the same time as islets, because of the immunosuppressive effects of MSCs^47–50^. Therefore, the transplantation of our IPCs with ADSCs may represent a new therapeutic strategy. Immunostaining of the transplanted NOD-IPCs showed that GAD65 expression increases with time, and the high expression of GAD65, which is a marker of autoimmune disease, may have promoted attacks on NOD-IPCs by CD8^+^ T cells. If so, approaches aimed at suppressing GAD65 expression might reduce the damage caused by CD8^+^ T cells. Petersen et al. reported that GABA transaminase inhibition reduces GAD expression in islets^51^. Interestingly, GAD65 was not expressed in the NOD-IPCs in vitro, and it is possible that valproic acid, which is a GABA-transaminase inhibitor used in our protocol, may have reduced the expression of GAD65. Thus, administration of a GABA transaminase inhibitor after transplantation may prolong the positive effect of IPC transplantation on BG concentration.

The limitations of the present study were that we principally used excised tissues and that cytokine concentrations and autoantibody titers were not measured. The assessment by immunohistochemical staining is not a quantitative evaluation, and thus it may lack objectivity. Especially, flow cytometry is an objective analysis for immune cells infiltrated into transplanted IPCs, and hence we plan to undertake further in vivo experiments by FACS analysis to increase the scientific validity. Moreover, we believe that clearer results could be obtained if isolated islets were transplanted as a positive control in the in vivo transplantation experiments. Therefore, further research is required to address these deficiencies. In addition, it is very important and interesting to assess the timing of MHC class I expression in our IPCs, considering autoimmune destruction after autotransplantation and allogeneic transplantation. We have started to investigate the timing of MHC class I and II expression in our generated IPCs (data not shown).

**Figure 5:**
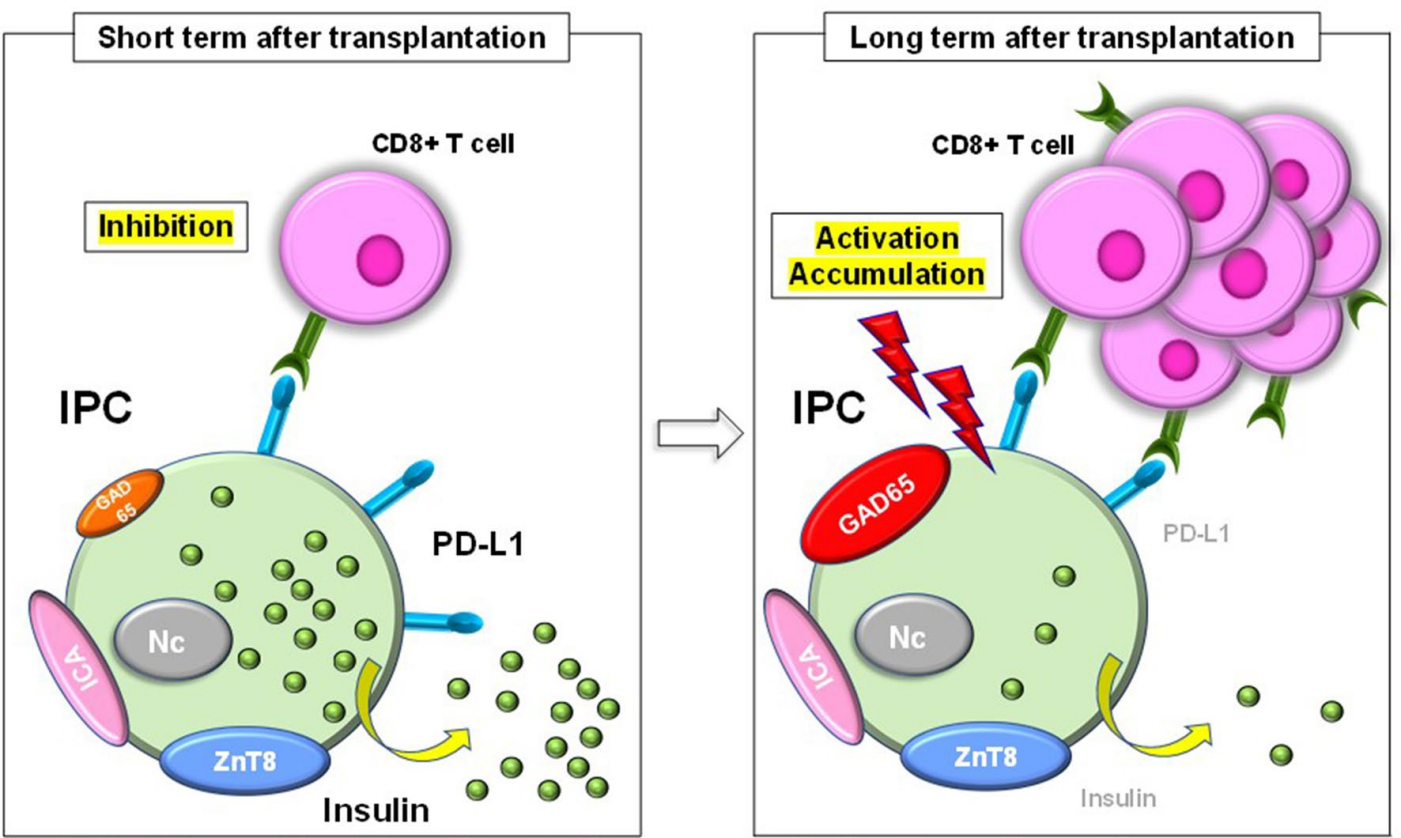
Proposed model for the autoimmune response to IPC transplantation in NOD mice with diabetes.

In conclusion, IPCs generated from ADSCs in the subcutaneous fat tissue of NOD mice express ICA and ZnT8, both of which are T1DM autoantigens. However, these cells also show high expression of PD-L1 in vitro. IPC transplantation results in a significant amelioration of diabetes in the short term, but after a longer period, T cells accumulate around the transplanted IPCs, which suggests that they are eventually recognized and damaged by the autoimmune responses that are characteristic of T1DM. Furthermore, the number of insulin and PD-L1-positive IPCs is significantly lower at this later time point. In addition, the expression of GAD65, a T1DM autoantigen, that is not expressed in vitro, gradually increases over time, which may promote attack by T cells. The present study presents the possibility that the immunological escape of the IPCs is temporary, and that they gradually become subject to damage by autoimmune responses. Therefore, it is necessary to develop strategies for the prevention of T1DM-related autoimmunity in the longer term, such as microencapsulation, enforcement of the generated cells, or the use of low doses of immunosuppressive agents. Here, we have characterized the life history of IPCs generated from ADSCs when transplanted into mice showing T1DM autoimmune responses (Fig. 5). This study has some limitations, such as the small number of samples, the lack of repeat in vivo experiments, and the inclusion of some preliminary data; however, it provides new insights into potential stem cell therapies for T1DM. In the future, therapeutic agents that upregulate the expression of PD-L1 in IPCs will be evaluated for their potential to improve the long-term survival of generated IPCs in recipients.

## Methods

### Mice

Twelve-week-old female NOD/ShiJcl mice were purchased from CLEA Japan, Inc. (Tokyo, Japan) and bred in an animal laboratory at Tokushima University under controlled light (12-h light/dark cycles) and temperature conditions. They had free access to water and standard chow. The present study was conducted in compliance with the Division for Animal Research Resources, Graduate School of Biomedical Sciences, Tokushima University (approval number: T2020-8). The experiments and procedures were approved by the Animal Care and Use Committee of the University of Tokushima and were performed in accordance with the NIH Guide for the Care and Use of Laboratory Animals. This study was carried out in compliance with the ARRIVE guidelines.

### Preparation of ADSCs

Adipose tissue was harvested from the inguinal region of NOD mice under general anesthesia in a sterile environment. The isolation of ADSCs was performed as previously described^52^. Briefly, the adipose tissue was stored in Hank’s Balanced Salt Solution (HBSS) and vigorously washed with HBSS. Then, the tissue was minced and digested with 2 mg/mL collagenase type IV (Thermo Fisher Scientific Inc., Waltham, MA, USA) for 120 min at 37 ℃ under gentle agitation. Dulbecco’s Modified Eagle Medium (DMEM; Life Technologies Japan Ltd., Tokyo, Japan) containing 10% fetal bovine serum (FBS; Life Technologies Japan Ltd.) was added to terminate the digestion. The tissue was filtered through a 100-μm cell strainer (Corning Inc., Corning, NY, USA), then centrifuged at 800 × *g* for 5 min. The cell pellet was washed with DMEM, then the cells were maintained in DMEM containing 10% FBS at 37 ℃ in a humidified 5% CO_2_ incubator. After approximately three passages, 2.0 × 10^6^ ADSCs were collected and mixed with 0.2 mg/mL recombinant RCP μ-pieces (Fujifilm, Tokyo, Japan), and then placed in the wells of a Nunchlon Sphera 96U Bottom Plate (Thermo Fisher Scientific Inc.).

### Differentiation protocol

NOD-IPCs were generated using a modification of our established protocol^5–10^ (Fig. 1a). Briefly, NOD-ADSCs were cultured in Step 1 (days 0–7) medium [DMEM/F12 (Thermo Fisher Scientific Inc.), 1% recombinant human albumin (Wako, Osaka, Japan), 10 nM exendin-4 (Sigma-Aldrich, St. Louis, MO, USA), 0.01% N2 supplement (Thermo Fisher Scientific Inc.), 0.01% B27 supplement (Thermo Fisher Scientific Inc.) and 50 ng/mL recombinant human activin-A (PeproTech Inc., Rocky Hill, NJ, USA)], then in Step 2 (days 8–21) medium, which was prepared in the same way as the Step 1 medium, but with the addition of 50 ng/mL recombinant human hepatocyte growth factor (PeproTech Inc.), valproic acid (Wako), and 10 mM nicotinamide (Sigma-Aldrich). Finally, from days 22 to 28, the IPCs were cultured in Step 2 medium containing 1% N2 and 1% B27 supplements. The culture media were changed every 2 days.

### FACS analysis

ADSCs (2.0 × 10^6^) were washed twice with phosphate-buffered saline (PBS) (Santa Cruz Bio., Dallas, TX, USA) and added to 100 μL of FACS buffer and 5 μL of antibodies targeting each of the following: CD31 (Thermo Fisher Scientific Inc.), CD34 (BioLegend, San Diego, CA, USA), CD45 (Thermo Fisher Scientific Inc.), CD90 (BioLegend), CD105 (Thermo Fisher Scientific Inc.), and CD146 (Thermo Fisher Scientific Inc.). After 30 min in a dark room at room temperature, the cells were analyzed using a FACSVerse and BD FACSuite software (BD Biosciences, San Jose, CA, USA).

### Dithizone staining

Cultured cells were stained with dithizone solution containing 50 mg of dithizone (Fujifilm Wako Pure Chemical Corporation, Tokyo, Japan) per 5 mL of dimethyl sulfoxide (Fujifilm Wako Pure Chemical Corporation). After three washes with PBS, IPCs were incubated in dithizone solution at 37 °C and 5% CO_2_. Stained samples were examined using the multi-purpose microscope, BZ-X710 (Keyence Engineering Corporation, Osaka, Japan), and BZ-X Analyzer (Keyence Software Corporation).

### Quantitative analysis of imaging

Cell images were digitally analyzed using Image J (National Institutes of Health, Bethesda, MD, USA) as previously reported^53^. Briefly, to analyze the dithizone staining, cell images were divided into three channels using the hue, saturation and value (HSV) model, and filtered using a hue threshold of 7 to 20. The mean gray saturation values at three random points in a spheroid area were measured in filtered images as the staining intensity. Dithizone staining intensity of highly functional IPCs was defined as > 180, as previously reported^8^. For the immunohistochemical analysis, sample images were filtered using a hue threshold of 20 to 28 after dividing images into three channels. The gray saturation at random points was measured on filtered images.

### Quantitative reverse transcription-PCR analysis

RNA was extracted from each sample using an RNeasy Mini Kit (Qiagen, Hilden, Germany) in accordance with the manufacturer’s instructions. Then, cDNA was synthesized using a reverse transcription kit (Applied Biosystems, Thermo Fisher Scientific Inc.). The primers for *Insulin* (Mm04207513_g1) and *Mafa* (Mm00845206_s1) from TaqMan gene expression assays were used. *Gapdh* (4352339E) was used as the reference gene. The StepOnePlus Real-Time PCR System (Applied Biosystems) was used to perform RT-qPCR.

### Western blotting

IPCs were collected and lysed using RIPA buffer (Thermo Fisher Scientific Inc.) supplemented with protease inhibitor cocktail (Sigma-Aldrich) and PhosSTOP phosphatase inhibitor cocktail (Roche, Tokyo, Japan). The protein concentrations were measured with a BCA assay kit (23,225; Thermo Fisher Scientific Inc.). Equal amounts of extracted protein samples were separated on 10% SDS-PAGE gels and transferred onto PVDF membranes (162–0177; Bio-Rad Inc., Hercules, CA, USA). The membranes were blocked with blocking buffer, and then incubated with the indicated primary antibody, followed by the appropriate horseradish peroxidase (HRP)-conjugated secondary antibody (#7074S; Cell Signaling Technology, Danvers, MA, USA). Protein bands were detected by chemiluminescence (Thermo Fisher Scientific Inc.). The primary antibodies used were anti-insulin antibody (ab181547, Abcam plc., Cambridge, UK), anti-PDX-1 antibody (#5679S; Cell Signaling Technology), and anti-β-actin (#4970S; Cell Signaling Technology).

### Measurement of glucose-stimulated insulin secretion

Glucose-stimulated insulin secretion was measured according to a previously described method^54,55^. Briefly, 3.2 × 10^5^ IPCs were pre-incubated for 2 h in 2 mL of RPMI medium (Wako, Osaka, Japan) supplemented with 0.5% bovine serum albumin (BSA; Sigma-Aldrich) and 2.2 mM glucose (Fujifilm Wako Pure Chemical Corporation). Thereafter, IPCs were incubated in RPMI medium containing 2.2 mM (basal) or 22 mM glucose for 1 h at 37 °C in a 5% CO_2_. After this, the supernatant of each sample was collected. The SI was calculated as the ratio of the insulin concentration after incubation in 22 mM glucose divided by the insulin concentration after incubation in 2.2 mM glucose.

### Enzyme-linked immunosorbent assay

Insulin concentration was measured using the LBIS Mouse Insulin ELISA Kit (Fujifilm Wako Shibayagi Corporation) and C-peptide concentration was measured using the LBIS Mouse C-peptide ELISA Kit (Fujifilm Wako Shibayagi Corporation) in accordance with the manufacturer’s protocols.

### Immunofluorescence staining

Immunofluorescence staining was performed as previously reported^10^. The following primary antibodies were used: anti-insulin (dilution 1:100, #4590; Cell Signaling Technology), anti-C-peptide (dilution 1:100, #4593S; Cell Signaling Technology), anti-PDX-1 (dilution 1:200, #5679; Cell Signaling Technology), anti-NKX6.1 (dilution 1:400, #54551; Cell Signaling Technology), anti-ICA (dilution 1:500, ab207750; Abcam plc.), anti-ZnT8 (dilution 1:500, 16169–1-AP, Proteintech, Chicago, IL, USA), anti-GAD65 (dilution 1:50, ab239372, Abcam plc.) and anti-PD-L1 (dilution 1:25, 17952–1-AP, Proteintech). Anti-rabbit Alexa 488 (A-11008; Thermo Fisher Scientific Inc.) for anti-C-peptide, PDX-1, ICA, ZnT8, GAD65 and PD-L1 antibodies, and anti-mouse Alexa 594 (A-11005; Thermo Fisher Scientific Inc.) for anti-insulin antibody were used as the secondary antibodies. 4′, 6-diamidino-2-phenylindole (DAPI) (P-36931; Thermo Fisher Scientific Inc.) was applied for nucleus staining.

### Immunohistochemical staining

Immunohistochemical staining was performed as previously reported^56^ with the following antibodies: anti-insulin (dilution 1:100, #4590; Cell Signaling Technology), anti-PDX-1 (dilution 1:50, #5679; Cell Signaling Technology), anti-PD-L1 (dilution 1:100, 17952–1-AP, Proteintech), anti-ICA (dilution 1:500, ab207750; Abcam plc.), anti-ZnT8 (dilution 1:500, 16169–1-AP, Proteintech), anti-GAD65 (dilution 1:2000, ab239372; Abcam plc.), anti-CD4 (dilution 1:1000, ab183685; Abcam plc.) and anti-CD8 (dilution 1:2000, ab217344; Abcam plc.).

### IPC transplantation

NOD mice that met the diabetic criterion were used as recipients. The criteria for diabetes were: two consecutive readings of > 350 mg/dL BG or one reading of > 400 mg/dL on a glucometer (Terumo Corporation, Tokyo, Japan). A total of 4.0 × 10^6^ IPCs were carefully collected and transplanted under the kidney capsule of the diabetic NOD mice under inhalation anaesthesia with isoflurane. IPC cell quantification was based on DNA content. As previously reported^7^, DNA content is largely preserved after IPC differentiation. Therefore, we estimated that there were about 2.0 × 10^4^ cells in one IPC cluster. All of the IPC clusters were of approximately the same size. Because 192 IPC clusters were transplanted into one NOD mouse, it was considered that approximately 4.0 × 10^6^ IPCs were transplanted. After transplantation, the BG of the mice was measured in tail-vein samples every 3 days. IPCs were transplanted into six NOD mice; two mice were killed for pathological investigations approximately 30 days after transplantation, and the remaining four NOD mice were observed with BG concentrations measured over 60 days. The measurable range of BG was 20 to 600 mg/dL.

### Statistical analysis

Data analysis was performed using JMP software version 13 (SAS, Cary, NC, USA). Comparisons between two groups were performed using the Mann–Whitney *U* test. Results are presented as mean ± standard deviation (SD). *P* < 0.05 was considered statistically significant.

## Supplementary Information

Supplementary Information 1.

## Data Availability

The datasets used and/or analyzed in this study are available from the corresponding author upon reasonable request.
