# Supplementary figures and images for: Syngeneically transplanted insulin producing cells differentiated from adipose derived stem cells undergo delayed damage by autoimmune responses in NOD mice
Source: Sci Rep. 2022 Apr 7;12:5852. doi: 10.1038/s41598-022-09838-x (PMC8991208; doi:10.1038/s41598-022-09838-x)

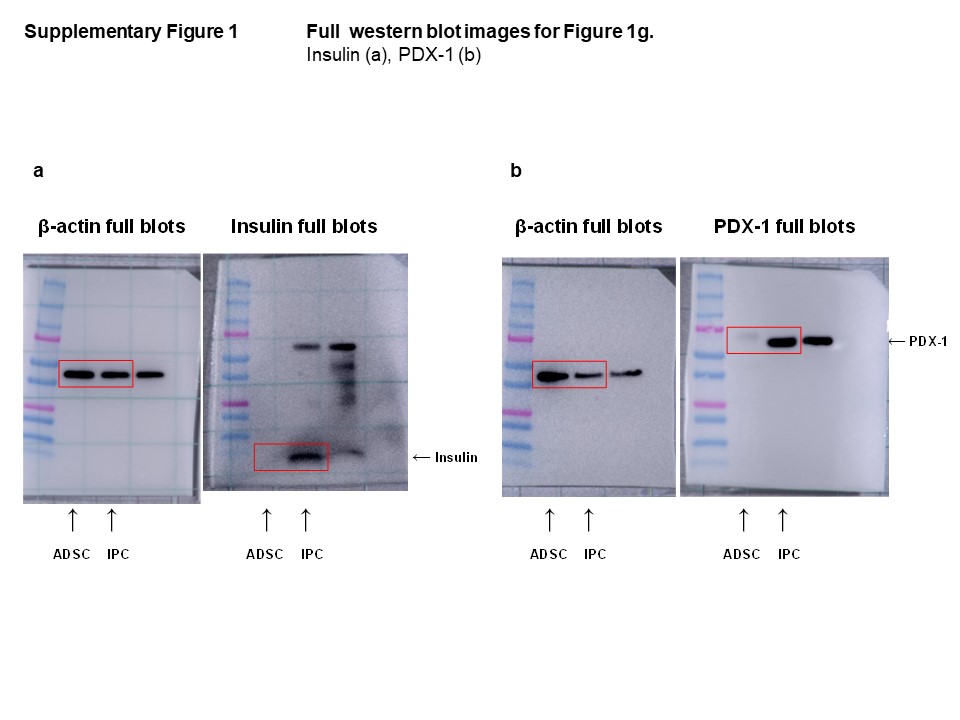

Supplement: Supplementary file 1 — Supplementary Information 1. [file 41598_2022_9838_MOESM1_ESM.jpg]
